# Adjunctive Chinese Herbal Products Therapy Reduces the Risk of Ischemic Stroke Among Patients With Rheumatoid Arthritis

**DOI:** 10.3389/fphar.2020.00169

**Published:** 2020-03-04

**Authors:** Hsuan-Shu Shen, Jen-Huai Chiang, Nai-Huan Hsiung

**Affiliations:** ^1^ Department of Chinese Medicine, Hualien Tzu Chi Hospital, Buddhist Tzu Chi Medical Foundation, Hualien, Taiwan; ^2^ School of Post-Baccalaureate Chinese Medicine, Tzu Chi University, Hualien, Taiwan; ^3^ Management Office for Health Data, China Medical University Hospital, Taichung, Taiwan; ^4^ College of Medicine, China Medical University, Taichung, Taiwan; ^5^ Department of Nursing, Asia University, Taichung, Taiwan

**Keywords:** rheumatoid arthritis, ischemic stroke, Chinese herbal products, National Health Insurance Research Database, traditional Chinese medicine

## Abstract

We performed a retrospective cohort study to investigate the association between the risk of ischemic stroke (IS) and the use of Chinese herbal products (CHP) in combination with western medicine (WM) among patients with rheumatoid arthritis (RA). The data were sourced from the registry for beneficiaries, inpatient and ambulatory care claims, and Registry for Catastrophic Illness from the National Health Insurance Research Database (NHIRD) in Taiwan between 1997 and 2011. Patients, who were newly diagnosed with RA between 1997 and 2010, were classified as the CHP group or non-CHP group depending on the presence of absence the adjunctive use of CHP following a diagnosis of RA. A total of 4,148 RA patients were in both the CHP and non-CHP groups after 1:1 matching. Patients in the CHP group had a significantly lower risk of IS compared to patients in the non-CHP group (adjusted hazard ratio [aHR], 0.67; 95% confidence interval [CI], 0.52–0.86). In the CHP group, patients who used CHP for more than 30 days had a lower risk of IS than their counterparts (aHR: 0.61, 95% CI: 0.40–0.91). Gui-Zhi-Shao-Yao-Zhi-Mu-Tang, Shu-Jin-Huo-Xie-Tang, and Du-Huo-Ji-Sheng-Tang might be associated with a lower risk of IS. Finally, the use of CHP in combination with WM was associated with a decreased risk of IS in patients with RA, especially among those who had used CHP for more than 30 days. A further randomized control trial is required to clarify the casual relationship between these results.

## Introduction

Patients with rheumatoid arthritis (RA) are at a greater risk of ischemic stroke (IS), which may be a leading cause of mortality and can lead to severe long-term disability (Benjamin et al., 2017). According to a meta-analysis study, the risk of stroke death was elevated by 52% among patients with RA (Avina-Zubieta et al., 2008). Danish and Taiwanese studies have also demonstrated that patients with RA were shown to have a 30% higher risk of IS compared to those without RA (Lindhardsen et al., 2012; Liou et al., 2014). Moreover, another prospective cohort study of 114,342 American women with RA found that the relative risk for IS was 1.48 (95% confidence interval [CI]:0.70–3.12) (Solomon et al., 2003). A possible mechanism might be the systemic inflammatory response in patients with RA who have elevated levels of C-reactive protein (CRP) and tumor necrosis factor-alpha (TNF)-α. This kind of chronic inflammation underlies the accelerated atherosclerosis, and is thought to be the main pathologic process of IS (Sattar et al., 2003; Hansson, 2005).

In addition to systemic chronic inflammation in individuals with RA, certain routinely prescribed medicines also increase the risk of IS (Nadareishvili et al., 2008; Lindhardsen et al., 2014). In accordance with the recommendations of the European League Against Rheumatism (EULAR), methotrexate, a kind of Disease-modifying antirheumatic drugs (DMARDs), plus short-term corticosteroids is the first-line therapy. When the treatment target cannot be achieved by methotrexate within 6 months or unfavorable prognostic factors are present, such as high disease activity, early erosion, or high level of rheumatoid factor, a TNF-antagonist may be considered as an add-on (Smolen et al., 2017). Previous research has documented that corticosteroids and certain non-steroidal anti-inflammatory drugs (NSAIDs) might increase the risk of IS in patients with RA 1.5- to 2-fold (Roubille et al., 2015). Methotrexate and TNF-antagonists, by contrast, are associated with the reduced risk of ischemic stroke (Tam et al., 2018). However, these drugs are associated with a potential risk of infection, fatigue, and liver damage (Salliot and van der Heijde, 2009). To avoid the side effects and debilitation associated with western medical treatment, the patients resorted to using traditional herbal remedies (Efthimiou and Kukar, 2010; Michalsen, 2013).

Thus, nearly 30% of individuals with RA were also taking Chinese herbal medicine in a recent study (Huang et al., 2015). Some of commonly used polyherbal formulations or single herbs have been demonstrated anti-inflammatory action, these include Gui-Zhi-Shao-Yao-Zhi-Mu-Tang (contains *Paeonia lactiflora* Pall., *Atractylodes macrocephala* Koidz., *Anemarrhena asphodeloides* Bge., *Saposhnikovia divaricata* (Turcz.) Schischk., *Glycyrrhiza uralensis* Fisch. ex DC., *Cinnamomum cassia* (L.) J.Presl, *Ephedra sinica* Stapf, *Aconitum carmichaelii* Debx., *Zingiber officinale* Roscoe) and *Boswellia neglecta* S.Moore (Ru-Xiang) (Ammon, 2006; Daily et al., 2017). Additionally, *Tripterygium wilfordii* Hook.f, *Stephania tetrandra* S Moore, and *Zingiber officinale* Roscoe have also been reported to have potential to suppress inflammation (Kang et al., 1996; Liacini et al., 2005; Wang et al., 2013). To date, most studies have purposely analyzed the effects of Chinese herbal products (CHP) in terms of alleviating the clinical symptoms and managing the chronic inflammation of patients with rheumatoid arthritis (Soeken et al., 2003; Zhang et al., 2011; Daily et al., 2017). However, there is a lack of studies that investigated whether the use of CHP was associated with a reduced risk of IS, which is a leading cause of death, in patients with RA. This study sought to evaluate the association between the use of CHP in combination with western medicine (WM) and the risk of IS in patients with RA by undertaking a large-scale retrospective cohort study.

## Materials and Methods

### Data Source

This study used reimbursement claims data from the Taiwan National Health Insurance Program. A national health insurance (NHI) program was implemented in March 1995, which covers 22.6 million individuals out of the total population of 23.0 million in Taiwan. The NHI is an obligatory universal health insurance program, which offers comprehensive medical care coverage to 99% of the entire Taiwanese population and has contracts with 97% of the hospitals and clinics there (http://www.nhi.gov.tw/english/index.aspx). The National Health Insurance Research Database (NHIRD) covers every medical record including Traditional Chinese medicine treatment that is reimbursed by the NHI.

The datasets used in the study consisted of a registry for beneficiaries, inpatient, and ambulatory care claims, and the Registry for Catastrophic Illness from the NHIRD. Ambulatory care claims contain the individuals’ gender, date of birth, date of visit, codes for the International Classification of Disease, Ninth Revision, and Clinical Modification (ICD-9-CM) codes for three primary diagnoses. Inpatient claims contain ICD-9-CM codes for principal diagnosis up to a total of four secondary diagnoses. The registry for Catastrophic Illness database contains data from insurers who suffer from major diseases and are granted exemption from co-payment. A disease diagnosis without valid supporting clinical findings may be considered a medical fraud by the NHI, which carries a penalty of 100-fold of the payment claimed by the treating physician or hospital. RA is a kind of major disease in Taiwan, and we can define patients with RA clearly from the Registry for Catastrophic Illness database. Because the NHIRD contains identified secondary data for research, the requirement for informed patient consent for the present study was waived off. This study was approved by the Institutional Review Board of the China Medical University (CMUH104-REC2-115). The datasets analyzed for the current study are available from the corresponding author on reasonable request.

### Selection of the Study Population

**Figure 1:**
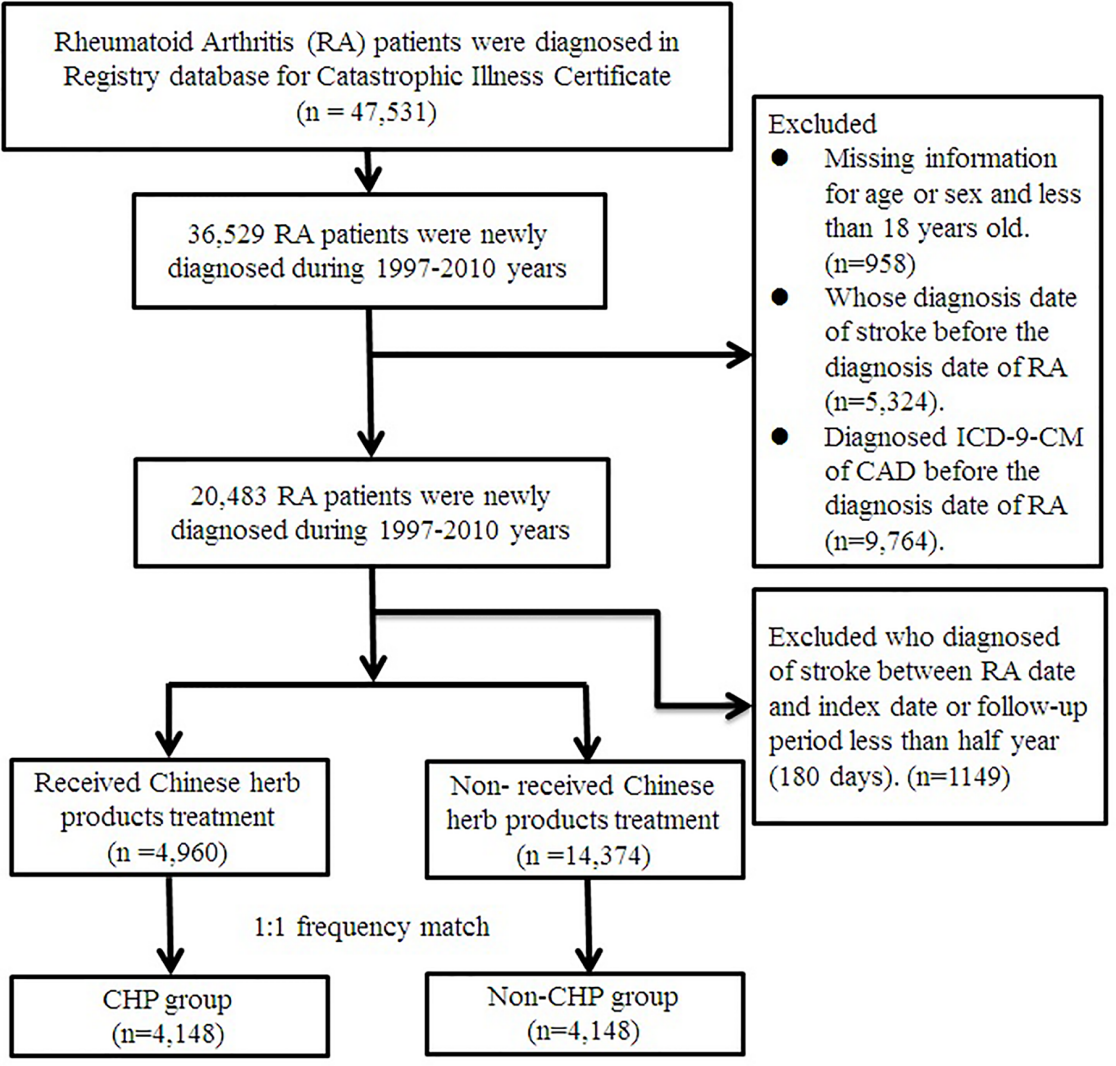
Flowchart of patients with rheumatoid arthritis. After excluding patients not fitting inclusion criteria, CHP and non-CHP groups comprised 4,148 patients after 1:1 matching. CHP, Chinese herbal products.

This was a retrospective cohort study. We identified patients who were diagnosed with RA (ICD-9-CM: 714.0) from the Registry for Catastrophic Illness database. Patients with RA who received WM treatment during the period from 1 January 1997 to 31 December 2010 were selected as the cohort group, and follow-up to 31 December 2011. The patients also had to be over the age of 18 years at diagnosis. The exclusion criteria were as follows: 1) dropout from insurance, 2) diagnosis of stroke (ICD-9-CM: 430-438) or coronary artery disease (ICD-9-CM: 410-414) before the first diagnosis date of RA, and 3) a follow-up period of less than 180 days. Finally, a total of 20,483 RA patients were included in the study (**Figure 1**).

The participants who had traditional Chinese Medicine outpatient visits along with CHP prescription records because of RA (ICD-9-CM code: 714.0) in combination with WM between the date of initial diagnosis of RA and the endpoint were defined as the CHP group. Those patients who only received WM because of RA were assigned to the non-CHP group. The date of the first accepted CHP after the diagnosis of RA was the index date for the CHP group. The index date of the patients in the non-CHP group was the first diagnosed date of RA. Prior to matching, there were 4,960 RA patients using CHP and 14,374 patients not using CHP. We used 1:1 frequency match by age (per 5 year-groups), gender, index year, and initial diagnosis year of RA. After frequency matching, there were 4,148 RA patients in both the CHP and non-CHP groups.

### Primary Outcome

The primary outcome was IS (ICD-9-CM: 433-438) during the 14-year follow-up with at least one inpatient claim and at least three ambulatory care claims. All eligible patients were followed up from the index date to December 31, 2011, initial diagnosis date of IS, date of withdrawal from NHI or date of death, whichever occurred first.

### Characteristics, Comorbidities, and Medication

The sociodemographic variables included age and gender. We identified comorbidities using ICD-9-CM from the database of outpatients and inpatients. Baseline comorbidity was defined as comorbid disease which occurred before the index date, and for which there were ambulatory care claims for at least 3 visits or at least one inpatient claim. Based on the recommendations of the EULAR, and hypertension (ICD-9-CM: 401-405), diabetes mellitus (ICD-9-CM: 250), hyperlipidemia (ICD-9-CM: 272) were considered as the covariates (Martin-Martinez et al., 2014; Agca et al., 2017). In addition, we also included baseline comorbidities which are the established risk factors for stroke, such as chronic obstructive pulmonary disease (ICD-9-CM: 491-496), end stage renal disease (ICD-9-CM: 585), and atrial fibrillation (ICD-9-CM: 427.31) as the covariates. We also considered drugs in accordance with the recommendations of the EULAR, including NSAIDs, corticosteroids, DMARDs, and TNF-antagonist prescribed after the initial diagnosis date of RA up to the endpoint of the study(Corticosteroid: beclometasone [ATC code: R03BA01], budesonide [ATC code: R03BA02], fluticasone [ATC code: R03BA05], ciclesonide [ATC code: R03BA08], formoterol [ATC code: R03AC13], and budesonide [ATC code: R03AK07]. DMARDs: tacrolimus [ATC code: L04AD02], mycophenolate mofetil [ATC code: L04AA06], azathioprine [ATC code: L04AX01], sulfasalazine [ATC code: A07EC01], hydroxychloroquine [ATC code: P01BA02], methotrexate [ATC code: L01BA01、L04AX03], leflunomide [ATC code: L04AA13], and cyclosporine [ATC code: L04AD01]. TNF-α antagonist: tanercept [ATC code: L04AB01], adalimumab [ATC code: L04AB04], rituximab [ATC code: L01XC02], and golimumab [ATC code: L04AB06]).

### Statistical Analysis

The difference of basic characteristics and potential confounders between the two groups was assessed by absolute standardized mean difference instead of statistical testing because the difference is a property of the sample rather than an underlying population. The value of absolute standardized mean difference ≤0.1 indicates a negligible difference in potential confounders between the two groups. A univariate and multivariate Cox’s proportional hazard model were used to estimate the hazard ratios (HRs) and 95% CI of IS for CHP use, controlling for potential confounding factors, including age, gender, diabetes mellitus, hypertension, hyperlipidemia, COPD, ESRD, atrial fibrillation, and medication. Furthermore, we performed a multivariate Cox’s proportional hazard model to estimate the hazard ratios (HRs) and 95% CI of IS for the most commonly prescribed single herb and polyherbal formulations controlling for age, gender, diabetes mellitus, hypertension, hyperlipidemia, COPD, ESRD, atrial fibrillation, and medication.

The Kaplan–Meier method and log rank tests were performed to compare the cumulative incidence rate of IS among the two groups. We also performed subsequent subgroup analyses to examine the effect of cumulative numbers of days of CHP use. We stratiﬁed the patients in the CHP group into three subgroups: cumulative numbers of days of CHP use days <30 days, 30 to 180 days, and >180 days. In this study, SAS 9.4 (SAS Institute Inc., Cary, NC) was used for the statistical analysis.

## Results

After the frequency matching, the gender and age were similar between the CHP and non-CHP groups. The mean (standard deviation) age of the patients in the CHP and non-CHP groups was 46.60 (11.59) years and 46.67 (11.59) years, respectively. There is no difference between the two group with regard to comorbidities (all of the standardized mean difference < 0.1). However, the proportion of DMARDs and TNF-antagonist use in the CHP group was significantly higher than that in the non-CHP group. The mean (median) follow-up period of the CHP group is longer than that of the non-CHP group (4.37(4.92) years v.s.4.81 (4.30) years, standardized mean difference = 0.108) (**Table 1**).

**Table 1:** Characteristics of patients with rheumatoid arthritis classified according to the use of Chinese herbal products.

Variable	Rheumatoid Arthritis				
	Accepted CHP			
	Non-CHP group (n = 4148)		CHP group (n = 4148)		Standardized mean difference*
	n	%	n	%
**Gender**					
Female	3591	86.57	3591	86.57	0.000
Male	557	13.43	557	13.43	0.000
**Age group**					
18–39	1157	27.89	1157	27.89	0.000
40–59	2468	59.5	2468	59.5	0.000
≥60	523	12.61	523	12.61	0.000
Mean ± SD (years)	46.67(11.59)		46.60(11.59)		0.006
**Baseline Comorbidity**					
Diabetes mellitus	340	8.2	288	6.94	0.047
Hypertension	810	19.53	706	17.02	0.065
Hyperlipidemia	447	10.78	469	11.31	0.017
COPD	540	13.02	682	16.44	0.097
ESRD	49	1.18	29	0.7	0.050
Atrial fibrillation	8	0.19	6	0.14	0.012
**Drug used**					
NSAID	4118	99.28	4136	99.71	0.061
Corticosteroid	163	3.93	203	4.89	0.047
DMARD	3906	94.17	4058	97.83	0.19
TNF-antagonist	625	15.07	1001	24.13	0.23
**Mean (median) of the follow-up period (years)**	4.81 (4.30)		5.37 (4.94)		0.108
**Duration between rheumatoid arthritis date and index, days (mean, median)**	942(616)		931(605)		0.012

*A value of standardized mean difference ≤0.1 indicates a negligible difference between the two groups.

CHP, Chinese herbal products; SD, standard deviation; COPD, chronic Obstructive Pulmonary Disease; ESRD, end stage renal disease; NSAID, non-steroidal anti-inflammatory drugs; DMARD, disease-modifying antirheumatic drugs; TNF, tumor necrosis factor.

**Figure 2:**
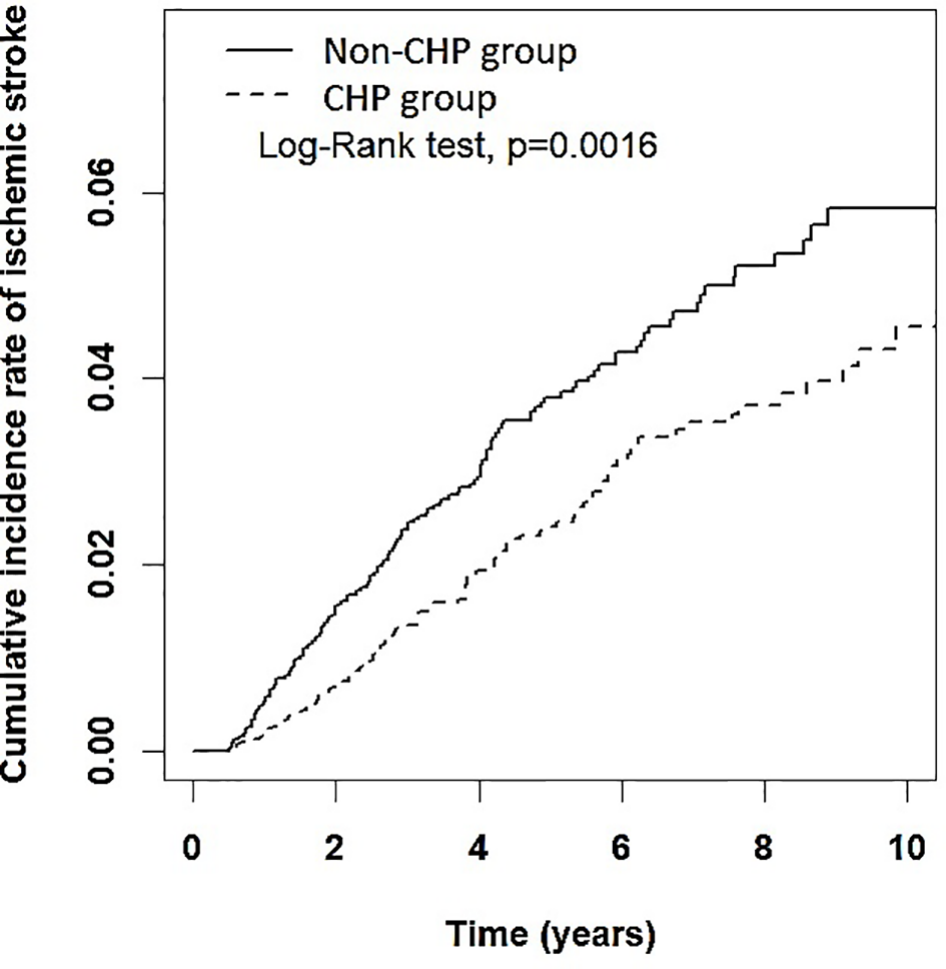
Cumulative incidence rate of ischemic stroke among patients with RA in the CHP and non-CHP group. CHP, Chinese herbal products.

A total of 250 patients developed IS during follow-up; the incidence rates of IS in the CHP and non-CHP group was 4.68 per 1000 person-years and 7.00 per 1000 person-years, respectively. The Kaplan–Meier analysis demonstrated that the cumulative incidence of IS in the CHP group was significantly lower than that in the non-CHP group (log-rank test, p = 0.0016) (**Figure 2**). Patients in the CHP group were more likely to have a lower risk of IS (crude HR: 0.67, 95% CI: 0.52–0.86) compared to their counterparts in the non-CHP group. After multivariate adjustment, the HR for the risk of IS remained the same (aHR: 0.67, 95% CI: 0.52–0.86) (**Table 2**).

**Table 2:** Incidence rates, hazard ratio, and confidence intervals of ischemic stroke among rheumatoid arthritis patients with and without Chinese herbal products usage according to gender, age, comorbidities, and drug used.

Variables	Rheumatoid Arthritis						Compared with non-CHP users	
	Non-CHP group (n = 4148)			CHP group (n = 4148)			Crude HR	Adjusted HR
	Event	Person years	IR^†^	Event	Person years	IR^†^	(95%CI)	(95%CI)
**Total**	146	20855	7.00	104	22211	4.68	0.67(0.52–0.86)**	0.67(0.52–0.86)**
**Gender**								
Female	122	18184	6.71	76	19208	3.96	0.59(0.44–0.79)***	0.63(0.47–0.85)**
Male	24	2671	8.99	28	3003	9.32	1.05(0.61–1.81)	1.35(0.74–2.44)
**Age group**								
18–39	9	6047	1.49	7	6306	1.11	0.74(0.27–1.98)	0.73(0.26–2.01)
40–59	96	12495	7.68	56	13331	4.20	0.55(0.39–0.76)***	0.63(0.45–0.89)**
≥60	41	2313	17.73	41	2574	15.93	0.89(0.58–1.38)	0.93(0.58–1.47)
**Baseline Comorbidity**								
Diabetes mellitus								
No	122	19274	6.33	90	20752	4.34	0.69(0.52–0.9)**	0.74(0.55–0.98)*
Yes	24	1581	15.18	14	1459	9.60	0.63(0.33–1.22)	0.64(0.32–1.31)
Hypertension								
No	94	17151	5.48	69	18848	3.66	0.67(0.49–0.91)*	0.7(0.5–0.96)*
Yes	52	3704	14.04	35	3363	10.41	0.74(0.48–1.13)	0.81(0.51–1.27)
Hyperlipidemia								
No	123	18908	6.51	81	20062	4.04	0.62(0.47–0.82)***	0.69(0.51–0.92)*
Yes	23	1947	11.82	23	2149	10.70	0.9(0.51–1.61)	0.95(0.52–1.74)
COPD								
No	122	18498	6.60	75	18854	3.98	0.6(0.45–0.8)***	0.71(0.52–0.95)*
Yes	24	2357	10.18	29	3357	8.64	0.86(0.5–1.48)	0.85(0.48–1.49)
ESRD								
No	142	20648	6.88	101	22072	4.58	0.67(0.52–0.86)**	0.73(0.56–0.95)*
Yes	4	207	19.35	3	139	21.54	1.04(0.23–4.64)	0.84(0.09–8.05)
Atrial fibrillation								
No	146	20838	7.01	104	22187	4.69	0.67(0.52–0.86)**	0.73(0.56–0.95)*
Yes	0	17	0.00	0	24	0.00	–	–
**Drug used**								
NSAID								
No	1	84	11.91	0	38	0.00	–	–
Yes	145	20771	6.98	104	22173	4.69	0.67(0.52–0.87)**	0.74(0.57–0.96)*
Corticosteroid								
No	134	20009	6.70	93	21019	4.42	0.66(0.51–0.86)**	0.73(0.55–0.96)*
Yes	12	846	14.18	11	1192	9.23	0.66(0.29–1.5)	0.59(0.24–1.43)
DMARD								
No	14	1189	11.78	4	537	7.45	0.63(0.21–1.93)	0.72(0.22–2.39)
Yes	132	19666	6.71	100	21674	4.61	0.69(0.53–0.89)**	0.73(0.56–0.96)*
TNF-α antagonist								
No	138	17579	7.85	87	16746	5.20	0.66(0.51–0.87)**	0.7(0.53–0.92)*
Yes	8	3276	2.44	17	5465	3.11	1.27(0.55–2.95)	1.04(0.44–2.48)

Adjusted HR: adjusted for CHP use, age, gender, diabetes mellitus, hypertension, hyperlipidemia, COPD, ESRD, atrial fibrillation, NSAID uses, corticosteroid, DMARD, and TNF-antagonist in Cox proportional hazards regression.

HR, hazard ratio; IR, incidence rates, per 1,000 person-years; CI, confidence interval; CHP, Chinese herbal products; COPD, chronic Obstructive Pulmonary Disease; ESRD, end stage renal disease; NSAID, non-steroidal anti-inflammatory drugs; DMARD, disease-modifying antirheumatic drugs; TNF, tumor necrosis factor.

*p < 0.05; **p < 0.01; ***p < 0.001.

After stratification by gender, the incidence rates of IS among the female and male patients in the CHP group were 3.96 per 1000 person-years and 9.32 per 1000 person-years, respectively. Female patients in the CHP group had a significantly lower risk of IS (aHR: 0.63, 95% CI: 0.47–0.85) compared to their counterparts in the non-CHP group. However, no significant difference was observed in this respect among the male patients (aHR: 1.35, 95% CI: 0.74–2.44) between the CHP and the non-CHP groups. In the age group of 40 to 59 years, the patients in the CHP group showed a significantly lower risk of IS compared to those in the non-CHP group (**Table 2**). In addition, RA patients without DM in the CHP group were associated with a lower risk of IS (aHR: 0.74, 95% CI: 0.55–0.98) compared with those in the non-CHP group. The same results were observed regarding the other baseline comorbidities, and RA patients without any comorbidity might be associated with a 0.73 to 0.69-fold lower risk of IS. Moreover, in the stratification analysis of the medication, RA patients receiving the CHP in combination with WM treatment showed a trend of reduced risk of IS compared with those receiving WM only, with the exception of RA patients taking TNF-antagonist (**Table 2**).

**Table 3** presents the results of the subgroup analysis according to the cumulative numbers of days of CHP use among patients with RA. Compared to the non-CHP group, patients who received CHP for 30 to 180 days had a significantly lower risk of IS (aHR: 0.61, 95% CI: 0.40–0.91). Moreover, patients who use CHP >180 days showed a marginal significant lower risk of IS among patients with RA (aHR: 0.62, 95% CI: 0.38–1.00).

**Table 3:** Hazard Ratios and 95% confidence intervals of ischemic stroke risk associated with the cumulative numbers of days of CHP use among patients with rheumatoid arthritis.

	n	Event	Hazard Ratio(95% CI)	
		no. (n = 250)	Crude	Adjusted^†^
**Non-CHP group**	4148	146	1(reference)	1(reference)
**CHP group**				
<30 days	1966	57	0.83(0.61–1.12)	0.88(0.64–1.20)
30–180 days	1380	28	0.53(0.35–0.79)**	0.61(0.40–0.91)*
>180 days	802	19	0.57(0.35–0.92)*	0.62(0.38–1.00)

Crude HR* represented relative hazard ratio;

Adjusted HR^†^ represented adjusted hazard ratio: mutually adjusted for age, gender, diabetes mellitus, hypertension, hyperlipidemia, COPD, ESRD, atrial fibrillation, NSAID uses, corticosteroid, DMARD, and TNF-antagonist in Cox proportional hazard regression.

HR, hazard ratio; CI, confidence interval; CHP, Chinese herbal products; COPD, chronic Obstructive Pulmonary Disease; ESRD, end stage renal disease.

*p < 0.05, **p < 0.01.

The five most commonly prescribed single herbs and polyherbal formulations are listed in **Table 4**. The most commonly used single herb was *Corydalis ambigua* Cham. & Schltdl. (Yan-Hu-Suo, n = 954, 22.9%), followed by *Spatholobus suberectus* Dunn (Ji-Xie-Teng, n = 791, 19.0%), *Cyperus rotundus* L. (Xiang-Fu, n = 675, 16.4%), *Morus alba* L. (Sang-Zhi, n = 741, 17.9%), and *Coix lacryma-jobi* L. (Yi-Yi Ren, n = 680, 16.39%). In addition, Gui-Zhi-Shao-Yao-Zhi-Mu-Tang (n = 1449, 34.9%) was the most commonly used polyherbal formulation, followed by Dang-Gui-Nian-Tong-Tang (n = 1182, 28.5%), Shu-Jin-Huo-Xie-Tang (n = 991, 23.9%), Du-Huo-Ji-Sheng-Tang (n = 696, 16.8%), and Jia-Wei-Xiao-Yao-San (n = 628, 15.1%). The association between prescribed polyherbal formulations and the risk of IS was explored using a Cox proportional hazard models. The results demonstrated that Gui-Zhi-Shao-Yao-Zhi-Mu-Tang (aHR: 0.50, 95% CI: 0.31-0.80), Shu-Jin-Huo-Xie-Tang (aHR: 0.35, 95% CI: 0.20-0.63), and Du-Huo-Ji-Sheng-Tang (aHR: 0.57, 95% CI: 0.34-0.96) were associated with a decreased risk of IS (**Table 4**).

**Table 4:** Hazard Ratios and 95% confidence intervals of ischemic stroke risk associated with the type of single herbs and polyherbal formulations used among patients with rheumatoid arthritis.

CHM prescription	Ischemic stroke		Hazard Ratio(95% CI)	
	n	No. of Event	Crude*	Adjusted^†^
**Non-CHP group**	4148	104	1(reference)	1(reference)
**CHP group**				
Single herb				
1. *Corydalis ambigua* Cham. & Schltdl. (Yen-Hu-So)	954	29	0.78(0.52–1.16)	0.92(0.61–1.38)
2. *Spatholobus suberectus* Dunn (Ji-Xie-Teng)	791	22	0.69(0.44–1.08)	0.81(0.52–1.29)
3. *Cyperus rotundus* L. (Pao-Fu-Tzu)	675	18	0.70(0.43–1.14)	0.87(0.53–1.44)
4. *Morus alba* L. (Sang-Chih)	741	21	0.71(0.45–1.12)	0.84(0.52–1.33)
5. *Coix lacryma-jobi* L. (Yi-Yi Jen)	680	19	0.71(0.44–1.14)	0.84(0.51–1.36)
Polyherbal formulation				
1. Gui-Zhi-Shao-Yao-Zhi-Mu-Tang	1449	21	0.44(0.28–0.70)***	0.50(0.31–0.80)**
2. Dang-Gui-Nian-Tong-Tang	1182	35	0.61(0.42–0.89)**	0.71(0.48–1.03)
3. Shu-Jin-Huo-Xie-Tang	991	13	0.32(0.18–0.56)***	0.35(0.20–0.63)***
4. Du-Huo-Ji-Sheng-Tang	696	16	0.54(0.32–0.91)*	0.57(0.34–0.96)*
5. Jia-Wei-Xiao-Yao-San	628	18	0.72(0.44–1.17)	1.06(0.64–1.76)

Crude HR^*^ represented relative hazard ratio; Adjusted HR^†^ represented adjusted hazard ratio: mutually adjusted for CHP use, age, gender, diabetes mellitus, hypertension, hyperlipidemia, COPD, ESRD, atrial fibrillation, NSAID, corticosteroid, DMARD, and TNF-antagonist usage in Cox proportional hazard regression.

CI, confidence interval; CHP, Chinese herbal products; COPD, chronic Obstructive Pulmonary Disease; ESRD, end stage renal disease; NSAID, non-steroidal anti-inflammatory drugs; DMARD, disease-modifying antirheumatic drugs; TNF, tumor necrosis factor.

*p < 0.05, **p < 0.01, ***p < 0.001.

## Discussion

This is the first nationwide retrospective cohort study designed to investigate the association between the risk of IS and the combined use of CHP in patients with RA. A total of 8,296 patients with RA were included in the study. There were 4,148 patients in each group after a 1:1 frequency match by gender, age, index year, and initial diagnosis year of RA. We observed an association between using CHP in combination with WM and a lower risk of IS among patients with RA (aHR for IS: 0.67 vs. non-CHP group). A possible reason for this is that the commonly used CHP, such as Gui-Zhi-Shao-Yao-Zhi-Mu-Tang, alleviate systemic inflammation by lowering the CRP, and erythrocyte sedimentation rate (ESR), which may decrease the progression of atherosclerosis, the main pathologic process of IS (Daily et al., 2017). Another commonly prescribed single herb, *Spatholobus suberectus* Dunn (Ji-Xie-Teng), has also been shown to suppress inflammation by decreasing TNF-α secretion (Ye et al., 2014). The findings of this study suggest that the RA patients had a decreased risk of IS which may be associated with the combined use of CHP with anti-inflammatory effects.

From the viewpoint of TCM, the etiology of RA is dampness, wind, and heat (23). In our study, Gui-Zhi-Shao-Yao-Zhi-Mu-Tang, which originated from the Synopsis of the Golden Chamber written by Zhang Zhong Jing in the Han Dynasty, was the most commonly prescribed polyherbal formulation (Bian, 2000). In TCM theory, Gui-Zhi-Shao-Yao-Zhi-Mu-Tang dispels dampness, eliminates wind, and clears heat, which is similar to the mechanism of anti-inflammatory drugs. Guo documented that Gui-Zhi-Shao-Yao-Zhi-Mu-Tang may partially attenuate RA by reversing inflammation-immune system imbalance and regulating the signaling pathways such as those involved in the secretion of TNF-α (Guo et al., 2016). Daily et al. noted that Gui-Zhi-Shao-Yao-Zhi-Mu-Tang lowered CRP and ESR in several clinical trials (Daily et al., 2017). Moreover, single herbs in Gui-Zhi-Shao-Yao-Zhi-Mu-Tang such as *Paeonia lactiflora* Pall. (Shao-Yao) and *Anemarrhena asphodeloides* Bunge (Zhi-Mu) have an anti-inflammatory effect. Compared with patients with RA who received leflunomide alone, those who received a combination of total glucosides of peony and leflunomide showed lower levels of the CRP, ESR, and rheumatoid factor (Feng et al., 2016). This may explain the lower risk of IS among patients with RA who received a combination of CHP and WM in the present study.

Interestingly, the study finding is that female patients in the CHP group had a significantly lower risk of IS [aHR:0.63 (95% CI: 0.47–0.85)] than those who were in the non-CHP group. However, no significant difference was observed in this respect for the in male patients. This result might be related to the patients’ lifestyle, such as tobacco use, which is a well-established risk factor for stroke. According to previous surveys of Taiwanese people, the rate of smoking was higher in males than in females (Wen et al., 2005). Additionally, smoking and obese male tend to engage in irregular exercise (Lin et al., 2016). This then establishes the factors for stroke that might influence this study’s results (Pearson et al., 2002; Boehme et al., 2017).

**Table 2** presents that there was no significant difference between the two groups in risk of IS among the RA patients with any kind of comorbidity which is the main risk factor for IS. However, a reduced risk of IS was observed in the CHP group among RA patients without any kind of comorbidity. A possible explanation for this is that the baseline comorbidities, major risk factors for IS, may reduce the efficacy of CHP and exert an influence on the risk of IS. Hypertension, diabetes, hyperlipidemia, chronic obstructive pulmonary disease, end stage renal disease, and atrial fibrillation are thought to be the independent risk factors for IS with 2- to 3-fold increased risk in stroke (Boehme et al., 2017). Possible mechanisms leading to stroke are chronic systemic inflammation (Austin et al., 2016; Chen R. et al., 2016; Jimenez et al., 2016; Nayak-Rao and Shenoy, 2017; Menet et al., 2018). Inflammation plays an important role in the development of the atherosclerotic plaque and the main pathologic process of IS. Although several commonly prescribed CHP have anti-inflammation effects, the chronic inflammation among RA patients with any kind of comorbidity might be too severe to be reversed. Therefore, there was no association between protective effect against IS of CHP and the risk of IS among RA patients with comorbidities. In the aspect of medication, RA patients receiving CHP in combination with DMARDs had a lower risk of IS compared to those receiving WM only. However, there is no significantly lower risk of IS among RA patients receiving CHP in combination with TNF-antagonist compared to those receiving WM only. The reason might be because the systemic inflammation of RA patients receiving TNF-antagonist are too severe to be alleviated. Doctors usually prescribe TNF-antagonist to treat RA patients due to high disease activity, early joints erosion, or high level of rheumatoid factor indicate higher grade of inflammation and disease severity than those taking DMARDs only (Albrecht and Zink, 2017). Hence, the association between a lower IS risk and receiving the CHP treatment in combination with WM among severe RA patients was not significant.

In the CHP group, patients with cumulative numbers of days of CHP use between 30 and 180 days were found to have a lower risk of IS than those whose cumulative numbers of days of CHP use <30 days. A possible explanation for this is that CHP requires 30-180 days to achieve a steady effect in patients with RA. The cumulative numbers of days of CHP use was similar to the treatment duration of methotrexate recommended by the European League Against Rheumatism (Smolen et al., 2017). Moreover, the results obtained for the minimum number of days of CHP use were consistent with those of previous randomized controlled trials. Patients with RA taking *Paeonia lactiflora* Pall. (Shao-Yao) extract in combination with treatment with leflunomide and methotrexate for 24 weeks had lower CRP levels and ESR (Chen et al., 2013). Additionally, after 12 to 24 weeks of treatment with *Tripterygium wilfordii* Hook.f in combination with methotrexate, there was an improvement in the level of CRP (Lv et al., 2015). Furthermore, several randomized controlled trial about the efficacy of Gui-Zhi-Shao-Yao-Zhi-Mu-Tang with WM showed that after taking Gui-Zhi-Shao-Yao-Zhi-Mu-Tang with WM for 4-12 weeks, there was a significant decrease in ESR, RA factor, and CRP (Daily et al., 2017). The Pathological mechanisms of RA are characterized by chronic systemic inflammation, and patients using CHP for 30-180 days may achieve an improved anti-inflammatory effect in terms of reducing their risk of IS.

Another major finding of this study is that the commonly prescribed polyherbal formulations, such as Gui-Zhi-Shao-Yao-Zhi-Mu-Tang, Shu-Jin-Huo-Xie-Tang, Du-Huo-Ji-Sheng-Tang, have been found to lower the risk of IS (**Table 4**). A possibly explanation is that the polyherbal formulations may suppress chronic inflammation in patients with RA. The function, ingredients, and the percentage of every single herb of the top 5 most commonly prescribed polyherbal formulations were presented in **Table 5**. Daily et al. have reported that Gui-Zhi-Shao-Yao-Zhi-Mu-Tang may lower the levels of the CRP and the ESR in patients with RA (Daily et al., 2017). Previous research has demonstrated that more than 50% of ingredients contained in Gui-Zhi-Shao-Yao-Zhi-Mu-Tang have anti-inflammatory effects with lowering the level of rheumatoid factor or suppressing the production of TNF-α or IL-6; these included *Paeonia lactiflora* Pall. (Bai-Shao), *Anemarrhena asphodeloides* Bge. (Zhi-Mu), *Saposhnikovia divaricata* (Turcz.) Schischk. (Fang-Feng) and *Zingiber officinale* Roscoe (Sheng-Jiang) (Chen et al., 2013; Kong et al., 2013; Funk et al., 2016). Although there was only a Chinese study presented that Shu-Jin-Huo-Xie-Tang may reduce the production of lymphocyte IL-2 after co-culture of the herb with lymphocyte. More than 50% of ingredients in Shu-Jin-Huo-Xie-Tang are demonstrated to have anti-RA effects, such as *Angelica sinensis* (Oliv.) Diels (Dang-Gui), *Ligusticum chuanxiong* Hort. (Chuan-Qiong), *Paeonia lactiflora* Pall. (Bai-Shao), *Atractylodes lancea* (Thunb.) DC. (Cang-Zhu), *Clematis chinensis* Osbeck (Wei-Ling-Xian), *Saposhnikovia divaricata* (Turcz.) Schischk. (Fang-Feng) and *Zingiber officinale* Roscoe (Sheng-Jiang), have been shown to suppress the expression of several inflammatory cytokines in an experimental RA model (Peng et al., 2012; Kong et al., 2013; Funk et al., 2016; Hung and Wu, 2016; Liu et al., 2016; Li et al., 2016). In addition, Du-Huo-Ji-Sheng-Tang has been claimed to mediate an anti-inflammatory effects by promoting lymphatic drainage function in TNF-Tg mice (Chen Y. et al., 2016). Certain single herbs in Du-Huo-Ji-Sheng-Tang also have an potentially anti-inflammatory effect, such as *Saposhnikovia divaricata* (Turcz.) Schischk. (Fang-Feng), these include *Paeonia lactiflora* Pall (Shao-Yao), *Ligusticum chuanxiong* Hort. (Chuan-Qiong), and *Angelica sinensis* (Oliv.) Diels (Dang-Gui). In previous studies, *Ligusticum chuanxiong* Hort. (Chuan-Qiong) and *Angelica sinensis* (Oliv.) Diels (Dang-Gui) have been demonstrated to counteract the expression of inflammatory cytokine thereby minimize the endothelial damage, such as TNF-α and interleukins, which is a key mediator of inflammation (Kong et al., 2013; Li et al., 2016; Hung and Wu, 2016). The anti-inflammatory effects of these polyherbal formulations may be associated with a lower risk of IS among patients with RA.

**Table 5:** The function and active compounds of the top 5 commonly prescribed single herbs and the function and ingredients of the top 5 most commonly prescribed polyherbal formulations for patients with RA.

Name	Active compounds		Function	Reference
**Single herb**				
Corydalis ambigua Cham. & Schltdl. (Yan-Hu-Suo)	Corynoline, Acetylcorynoline,d-corydalin, dl-tetrahydropalmatine, Protopine, Tetrahydrocoptisine, dl-tetrahydroCoptisine, d-corybulbine, Allocryptopine		Pain tolerance↑ Anti-hypertension Anti-inflammation	(Zhou et al., 2016; Zhou et al., 2019)
*Spatholobus suberectus* Dunn (Ji-Xie-Teng)	Jixuetengstero, Friedelin, Friedelinsterol, Taraxerone, β- Sitosterol, Stigmasterol, Campesterol		Anti-inflammation Anti-oxidant effects Anti-viral effects Immunomodulatory	(Fu et al., 2017; Pang et al., 2011)
*Cyperus rotundus* L. (Xiang-Fu)	α-longipinane, β-selinene, Cyperene, Caryophyllene oxide		Anti-nociceptive effects Anti-inflammatory Anti-oxidant effects Anti-lipidemic,	(Imam and Sumi, 2014; Pirzada et al., 2015)
*Morus alba* L. (Sang-Zhi)	Morin, Oxyresveratrol, Mulberrin, Mulbel- Cochromene, Cyclomulberrin, Cyclomulbel-rochromene		Anti-inflammation Anti-oxidant Anti-tumour activities Anti-hyperlipidemic,	(Bachewal et al., 2018; Park et al., 2016; Li et al., 2015)
*Coix lacryma-jobi* L. (Yi-Yi-Ren)	Coixol, Coixenolide, Myristic acid		Anti-inflammation Anti-oxidant effects Anti-tumour activities	(Seo et al., 2000)

**Name**	**Ingredients**	**Percentage (%)**	**Function**	**Reference**
**Polyherbal formulation**				
Gui-Zhi-Shao-Yao-Zhi-Mu-Tang	*Paeonia lactiflora* Pall. (Bai-Shao) *Atractylodes macrocephala* Koidz. (Bai-Zhu) *Anemarrhena asphodeloides* Bge. (Zhi-Mu) *Saposhnikovia divaricata* (Turcz.) Schischk. (Fang-Feng) *Glycyrrhiza uralensis* Fisch. ex DC. (Gann-Cao) *Cinnamomum cassia* (L.) J.Presl (Gui-Zhi) *Ephedra sinica* Stapf (Ma-Huang) *Aconitum carmichaelii* Debx. (Fu-Zi) *Zingiber officinale* Roscoe (Sheng-Jiang)	**9.6%** **16.1%** **12.9%** **12.9%** **6.5%** **12.9%** **6.5%** **6.5%** **16.1%**	Morning stiffness **↓** CRP**↓** RSR**↓** RA factor**↓**	(Efthimiou and Kukar, 2010; Ye et al., 2014)
Dang-Gui-Nian-Tong-Tang	*Artemisia capillaris* Thunb. (Yin-Chen) *Notopterygium incisum* K.C.Ting ex H.T.Chang (Qiang-Huo) *Saposhnikovia divaricata* (Turcz.) Schischk. (Fang-Feng) *Actaea heracleifolia* (Kom.) J.Compton (Sheng-Ma) *Pueraria lobata* (Willd.) Ohwi (Ge-Gen) *Atractylodes lancea* (Thunb.) DC. (Cang-Zhu) *Atractylodes macrocephala* Koidz. (Bai-Zhu) *Glycyrrhiza uralensis* Fisch. ex DC. (Gan-Cao) *Scutellaria baicalensis* Georgi (Huang-Qin) *Sophora flavescens* Ait. (Ku-Shen) *Anemarrhena asphodeloides* Bge. (Zhi-Mu) *Angelica sinensis* (Oliv.) Diels (Dang-Gui) *Polyporus umbellatus* (Pers.) Fries (Zhu-Ling) *Alisma plantago-aquatica* subsp. *orientale* (Sam.) Sam. (Ze-Xie) *Atractylodes lancea* (Thunb.) DC	10.6% 10.6% 6.0% 4.6% 4.6% 4.6% 6.0% 10.6% 10.6% 4.6% 6.0% 4.6% 6.0% 6.0% 4.6%	Not available	
Shu-Jin-Huo-Xie-Tang	*Angelica sinensis* (Oliv.) Diels (Dang-Gui) *Ligusticum chuanxiong* Hort. (Chuan-Qiong) *Paeonia lactiflora* Pall. (Bai-Shao) *Rehmannia glutinosa* Libosch. (Di-Huang) *Atractylodes lancea* (Thunb.) DC. (Cang-Zhu) *Cyathula officinalis* Kuan (Niu-Xi) *Citrus reticulata* Blanco (Chen-Pi) *Prunus persica* (L.) Batsch (Tao-Ren) *Clematis chinensis* Osbeck (Wei-Ling-Xian) *Stephania tetrandra* S. Moore (Fang-Ji) *Notopterygium incisum* K.C.Ting ex H.T.Chang (Qiang-Huo) *Angelica dahurica* (Fisch. ex Hoffm.) Benth. et Hook. (Bai-Zhi) *Gentiana scabra* Bunge (Long-Dan -Cao) *Zingiber officinale* Roscoe (Sheng-Jiang) *Poria cocos* (Schw.) Wolf (Fu-Ling) *Saposhnikovia divaricata* (Turcz.) Schischk. (Fang-Feng) *Glycyrrhiza uralensis* Fisch. ex DC. (Gan-Cao)	7.2% 3.7% 9.1% 7.2% 7.2% 7.2% 7.2% 3.7% 7.2% 3.7% 3.7% 3.7% 3.7% 10.8% 3.7% 7.2% 3.7%	Interleukin 2 production **↓** Anti-hypersensitivity	(Imam and Sumi, 2014)
Du-Huo-Ji-Sheng-Tang	*Taxillus chinensis* (DC.) Danser (Sang-Ji-Sheng) *Angelica pubescens* Maxim. (Du-Huo) *Eucommia ulmoides* Oliv. (Du-Zhong) *Cyathula officinalis* Kuan (Niu-Xi) *Asarum sieboldii* Miq. (Xi-Xin) *Glycyrrhiza uralensis* Fisch. ex DC. (Gan-Cao) *Gentiana macrophylla* Pall. (Qin-Jiao) *Poria cocos* (Schw.) Wolf (Fu-Ling) *Cinnamomum cassia* Presl (Rou-Gui) *Saposhnikovia divaricata* (Turcz.) Schischk. (Fang-Feng) *Ligusticum chuanxiong* Hort. (Chuan-Qiong) *Rehmannia glutinosa* Libosch. (Di-Huang) *Paeonia lactiflora* Pall. (Bai-Shao) *Angelica sinensis* (Oliv.) Diels (Dang-Gui) *Panax ginseng* C. A. Mey. (Ren-Shen)	6.5% 9.0% 6.5% 6.5% 6.5% 6.5% 6.5% 6.5% 6.5% 6.5% 6.5% 6.5% 6.5% 6.5% 6.5%	Anti-inflammation	(Kong et al., 2013)
Jia-Wei-Xiao-Yao-San	*Glycyrrhiza uralensis* Fisch. ex DC. (Gan-Cao) *Paeonia lactiflora* Pall. (Bai-Shao) *Angelica sinensis* (Oliv.) Diels (Dang-Gui) *Poria cocos* (Schw.) Wolf (Fu-Ling) *Atractylodes macrocephala* Koidz. (Bai-Zhu) *Bupleurum chinense* DC. (Chai-Hu) *Mentha haplocalyx* Briq. (Bo-He) *Zingiber officinale* (Willd.) Rosc. (Gan-Jiang) *Moutan officinalis* (L.) Lindl. & Paxton (Mu-Dan-Pi) *Gardenia jasminoides* J.Ellis (Zhi-Zi)	6.1% 12.1% 12.1% 12.1% 12.1% 12.1% 6.1% 12.1% 7.6% 7.6%	Prevent bone loss Prevent atherosclerosis	(Pirzada et al., 2015)

This study has several strengths. First, this is a nationwide population-based study using a registry of beneficiaries, inpatient, and ambulatory care claims, and the Registry for Catastrophic Illness from the NHIRD datasets. This type of data not only prevents selection bias and recall bias related to the use of CHP but also reflects real-world practice. Second, the use of the date of first accepted CHP as the index date in the CHP group helps to avoid bias whereby patients with a longer stroke-free period of time tend to use CHP. Third, we performed a subgroup analysis based on the number of cumulative days of CHP usage. The result may provide empirical evidence pertaining to the days of CHP usage for clinical use.

Several limitations of the present study need to be considered. Data pertaining to life style, smoking, drinking, obesity, and body mass index are unavailable from the NHIRD. Similarly, data pertaining to disease activity and disease severity were unavailable, although all of them are known risk factors for IS (Dhillon and Liang, 2015). We performed a 1:1 frequency match and used a Cox proportional hazard model to eliminate the potential effect of confounding factors. Second, NHIRD only covers CHP prescribed by licensed TCM physicians. Patients who resorted to over-the-counter use of CHP may not have been included in the study sample, which is likely to have led to an underestimation of the percentage of CHP usage. Third, safety of prescribed polyherbal formulations are not available. Certain single herbs contained in the polyherbal formulations were reported to cause adverse reactions. *Aconitum carmichaelii* Debx.(accounts for 6.5%), contained in Gui-Zhi-Shao-Yao-Zhi-Mu-Tang, might cause palpitation, hypotension, and arrhythmia due to aconitine alkaloids. *Asarum sieboldii* Miq.(accounts for 6.5%), one of the single herb in the Du-Huo-Ji-Sheng-Tang, might lead to renal failure, tubulointerstitial fibrosis, and urothelial cancer owing to aconitine alkaloids. Although, the toxic compounds of the certain single herbs might result in harmful effects, Daily and Hsieh reported that no severe adverse events and drug reactions are observed after taking Gui-Zhi-Shao-Yao-Zhi-Mu-Tang and Du-Huo-Ji-Sheng-Tang 4 to 20 weeks (Hsieh et al., 2010; Daily et al., 2017).

## Conclusion

This study demonstrated the association between a decreased IS risk and receiving CHP treatment in combination with WM in RA patients, particularly those who used CHP for more than 30 days. Our findings suggest that Gui-Zhi-Shao-Yao-Zhi-Mu-Tang, Shu-Jin-Huo-Xie-Tang, and Du-Huo-Ji-Sheng-Tang might be associated with a lower risk of IS. A further randomized control trial is required to clarify the casual relationship between these results.

## Data Availability Statement

The datasets generated for this study are available on request to the corresponding author.

## Author Contributions

H-SS was responsible for the study’s conception and design, reviewing and interpreting the data, and drafting the manuscript. N-HH was responsible for modifying the study design, interpreting the data, and the critical revision. J-HC contributed to the collection and analysis of the data. H-SS, N-HH, and J-HC were responsible for the final approval of the revised manuscript. All authors read and approved the final manuscript.

## Conflict of Interest

The authors declare that the research was conducted in the absence of any commercial or financial relationships that could be construed as a potential conflict of interest.

The handling editor and reviewer RH declared their involvement as co-editors in the Research Topic, and confirm the absence of any other collaboration.
